# Predictability of the Ningaloo Niño/Niña

**DOI:** 10.1038/srep02892

**Published:** 2013-10-08

**Authors:** Takeshi Doi, Swadhin K. Behera, Toshio Yamagata

**Affiliations:** 1 Research Institute for Global Change (RIGC), Japan Agency for Marine-Earth Science and Technology (JAMSTEC), Yokohama, Japan; 2 Application Laboratory/JAMSTEC, Yokohama, Japan

## Abstract

The seasonal prediction of the coastal oceanic warm event off West Australia, recently named the Ningaloo Niño, is explored by use of a state-of-the-art ocean-atmosphere coupled general circulation model. The Ningaloo Niño/Niña, which generally matures in austral summer, is found to be predictable two seasons ahead. In particular, the unprecedented extreme warm event in February 2011 was successfully predicted 9 months in advance. The successful prediction of the Ningaloo Niño is mainly due to the high prediction skill of La Niña in the Pacific. However, the model deficiency to underestimate its early evolution and peak amplitude needs to be improved. Since the Ningaloo Niño/Niña has potential impacts on regional societies and industries through extreme events, the present success of its prediction may encourage development of its early warning system.

In austral summer of 2010/11, an unprecedented oceanic warm event was observed off the west coast of Australia. Sea surface temperature (SST) anomaly averaged in February-March 2011 reached about 3°C off the west coast of Australia, which is above four times of the standard deviation of its interannual variation in recent 30 years^1^. This coastal phenomenon was named the Ningaloo Niño^1^ in analogy of the Benguela Niño in the Atlantic^2^,^3^. This term is also based on the similarity between the coastal warm event and the equatorial warm event like El Niño in the Pacific^4^,^5^. The Ningaloo Niño is reported to have severe impacts on marine ecosystem of fishery and coral reef there^6^,^7^,^8^. It is also expected that this coastal warm event may have influences on the Australian summer precipitation through induced anomalous regional atmospheric circulations. Because of its potential impact on regional societies and industries, the Ningaloo Niño could be a topic of substantial research in seasonal prediction at a regional scale.

Study on the mechanism of the Ningaloo Niño has just started. Feng et al.^1^ have demonstrated that the 2011 warm event of the Ningaloo Niño was mostly driven by the oceanic heat transport of the poleward-flowing Leeuwin Current in austral summer. The unusual intensification of the Leeuwin Current was interpreted by remote forcing through oceanic and atmospheric routes traced back to the 2010/11 La Niña event in the tropical Pacific. Yamagata^9^ has recently presented the evolution process of the canonical Ningaloo Niño based on all significant events in the past 50 years, and has shown existence of two types of the Ningaloo Niño; some events are associated remotely with the La Niña events as discussed by Feng et al.^1^, but others are more locally excited. Interestingly, those two types of the Ningaloo Niño appear to be related to different conditions of the continental sea level pressure (SLP) modulated by the Australian summer monsoon and/or the Southern Annular Mode.

Together with efforts to understand the mechanism of the Ningaloo Niño/Niña, exploring its prediction is important. Hendon and Wang^10^ have shown that the Leeuwin Current can be predicted at 4–9 months ahead by use of the empirical downscaling method based on dynamical model forecast. In this paper, we first explore seasonal predictability of the Ningaloo Niño/Niña events over 30 years and then focus on the particular event of 2010/11 by use of a seasonal prediction system based on an ocean-atmosphere coupled general circulation model (CGCM) called SINTEX-F1^11^,^12^. The present work opens a door to predicting interannual variability of a regional climate mode in mid-latitudes.

## Results

### Ningaloo niño index

**Figure 1:**
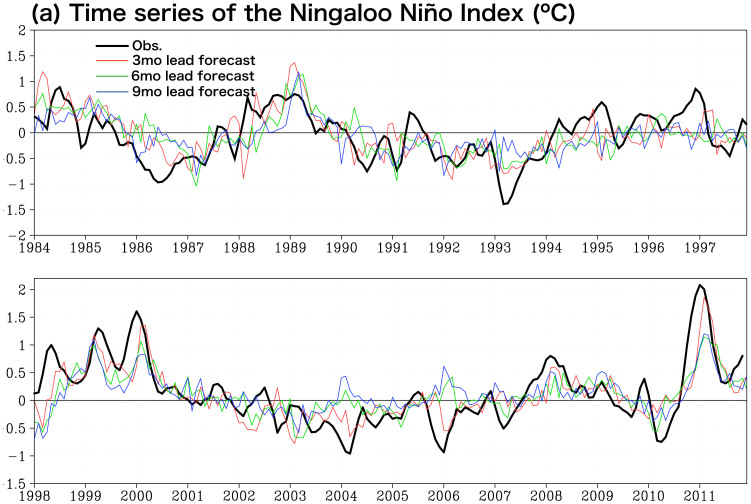
Three months averaged time series along a fixed start time of the Ningaloo Niño Index (NNI: SST anomalies averaged in 108°–116°E, 28°–22°S) for 3, 6, and 9 months lead prediction by the SINTEX-F1 and observational data of NOAA OISSTv2 (°C).

**Figure 2:**
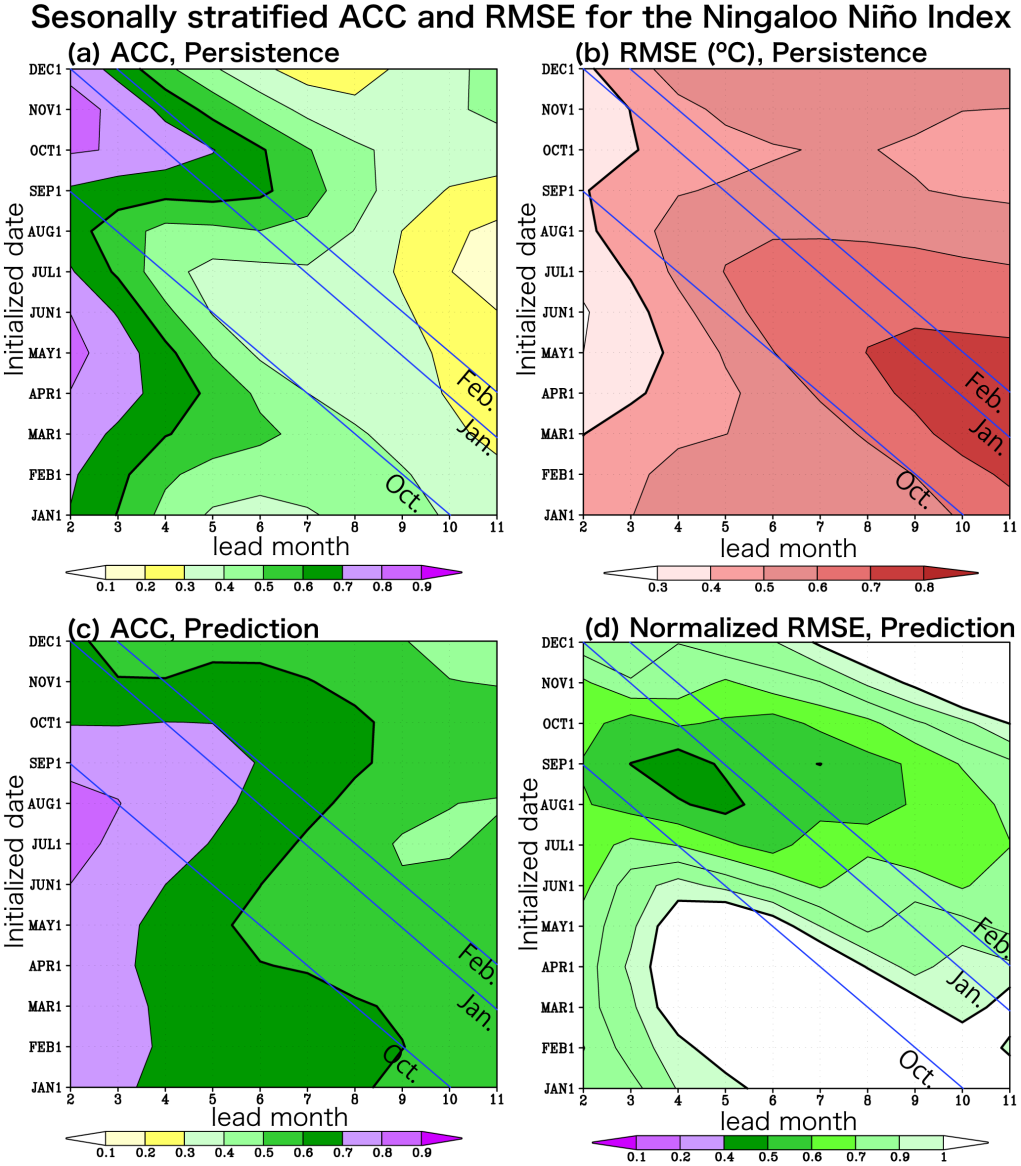
(a) Seasonally stratified anomaly correlation coefficients (ACC) of the persistence (lag auto-correlation of observation) for three months averaged Ningaloo Niño Index along a fixed start time in 1984–2011. Value of 0.6 is shown by thick black line. (b) Seasonally stratified route mean square errors (RMSE) of the persistence for the NNI in 1984–2011 (°C). Value of 0.4 is shown by thick black line. (c) Same as (a), but for SINTEX-F1 prediction. (d) Same as (b), but for SINTEX-F1 prediction normalized by the seasonal standard deviation of the observed Ningaloo Niño Index. Value of 0.5 is shown by thick black line.

**Figure 3:**
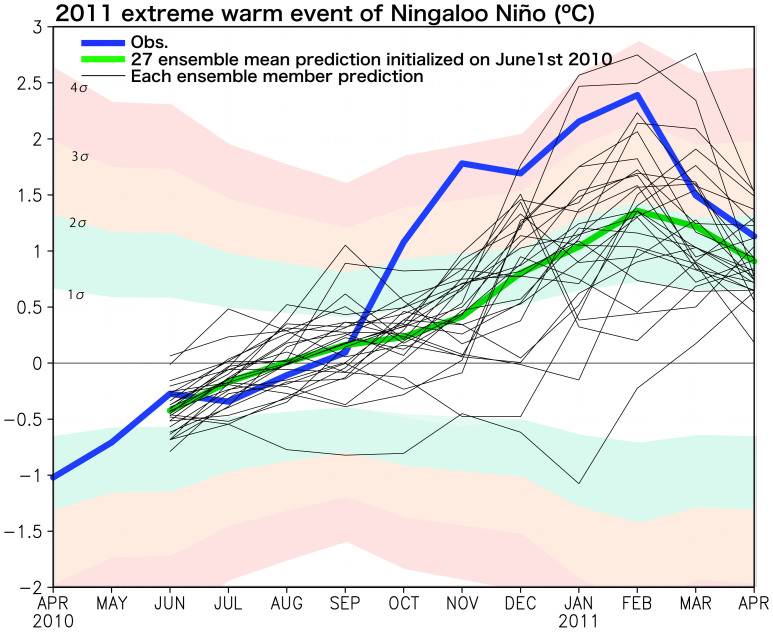
Monthly time series of the Ningaloo Niño Index (NNI) for observational data of NOAA OISSTv2 (blue) and SINTEX-F1 27 ensemble member prediction initialized on June 1st, 2010 in °C (ensemble member mean: green; each member: thin black). One, twice, three-, and four-times of the standard deviation of the observed NNI in 1983–2006 (σ) are also shown by shaded.

We begin by exploring historical time series of the Ningaloo Niño Index (NNI), which is defined as SST anomalies off the west coast of Australia (108°–116°E, 28°–22°S) on a basis of the first empirical orthogonal function (EOF) mode in the monthly SST anomaly off Western Australia (100–120°E, 36–14°S), which explains about 50% of the total variance. At a first glance, the SINTEX-F1 CGCM seems to be skillful in predicting most of significant events at least one season ahead, though the onset stage and the amplitude of the events are not well predicted (Fig. 1). Figure 2 shows seasonally stratified anomaly correction coefficients (ACC) and route mean square errors (RMSE). The SINTEX-F1 is skillful in predicting the Ningaloo Niño (ACC > 0.6 and normalized RMSE < 1) up to 5 months lead when it is initialized on the first day of each month from June to November. In particular, it predicts the Ningaloo Niño very well when it is initialized on the first day of each month from August to October. The ACC is larger than 0.6 above persistence up to 7 months lead with about 0.6 of the normalized RMSE when the model is initialized on August 1st. The 6-month lead prediction skill gradually rises and reaches the peak for October 1st initialization (ACC > 0.6 and normalized RMSE < 0.7 up to 8 months lead). Then, the skill suddenly drops for December 1st initialization. The prediction skill is lowest when initialized on the first day of each month from March to May. This seasonality of the prediction skill is due to the seasonal phase-locking nature of the Ningaloo Niño; it develops rapidly from October, reaches its peak in January-February, and decays gradually (e.g. Fig. 3).

### 2011 warm event

Here we focus on the extreme warm event in early 2011 (see Fig. 1). The SINTEX-F1 prediction initialized on June 1st, 2010 reasonably predicted the extreme warm event in early 2011 (Fig. 3, Table 1). The observed NNI in February 2011 was 2.4°C, which is above three times of the standard deviation observed for 1983–2011 period. The prediction from June 1st, 2010 showed 85% (44%) chance of a warm event above one (two) standard deviation in February 2011. However, it showed only 11% chance of an extreme warm event above three standard deviation. We may conclude that the 2011 warm event is predictable from June 1st, 2010 except for its early evolution and peak amplitude. In February 2011, the model predicted a peak anomaly of 1.4°C, which is almost half of the observed amplitude. The observation shows the rapid development of the Ningaloo Niño in October-November 2010 at a growth rate of 0.9°C/month, while the model predicted much slower rate of 0.15°C/month.

**Figure 4:**
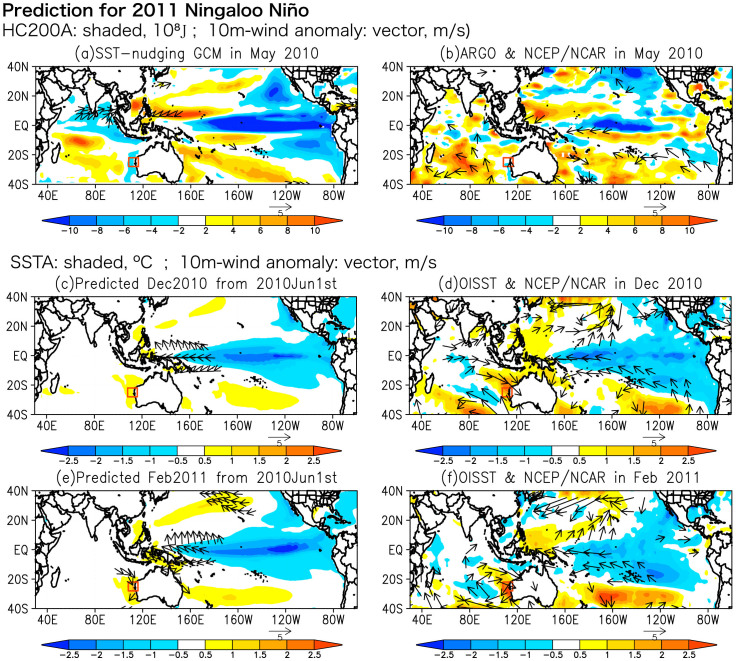
The SINTEX-F1 predicted field and observation in May, December 2010, and February 2011. (a) The SST-nudging SINTEX-F1 outputs for ocean heat content anomalies above a depth of 200 m (10^8^ J: shaded) and 10-m surface wind speed anomalies (m s^−1^: vector shows value beyond 2 m s^−1^) in May 2010. (b) Same as (a), but for observation of ARGO data for ocean heat content and NCEP/NCAR reanalysis data for wind speed. (c) SST anomalies (°C: shaded) and 10-m surface wind speed anomalies (m s^−1^: vector shows value above 2 m s^−1^) in December 2010 predicted by the model when initialized on June 1st, 2010. (d) Same as (c), but for observation of NOAA OISSTv2 for SST and NCEP/NCAR reanalysis data for wind speed. (e) Same as (c), but for February 2011. (f) Same as (d), but for February 2011. The GrADS software was used for this figure.

To examine details of the evolution, horizontal maps of both predicted and observed SST and surface wind anomalies are shown in Figs. 4c–f. In May 2010, the preconditioning phase of the 2011 warm event, the Indian Ocean basin was warmer-than-normal owing to the 2009/10 El Niño event by the so-called capacitor effect^13^, which induces easterly wind anomalies in the equatorial western Pacific, leading to the quick transition from El Niño to La Niña. We note that the observed SST anomalies were used to initialize the model prediction. As shown in the ocean heat content anomalies above a depth of 200 m (Figs. 4a and b), the model reasonably captured the La Niña condition in the Pacific as well as the easterly wind anomalies in the equatorial western Pacific^1^. Also, the SINTEX-F1 captured the warm heat content anomalies in the tropical southern Indian Ocean and the tropical western Pacific, which were consistent with the observation. However, the model simulated the cold heat content anomalies in the northern tropical Indian Ocean, although the SST anomaly was warm there. Despite the disagreement in the northern Indian Ocean, the SINTEX-F1 captured the significant feature of the subsurface ocean condition in May 2010.

**Figure 5:**
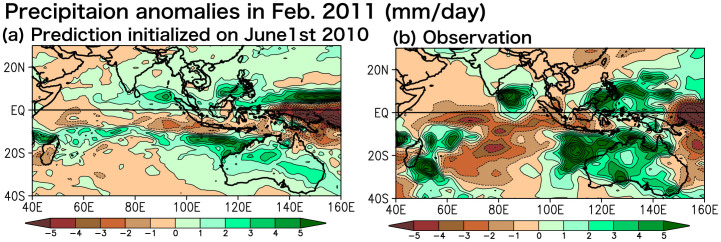
(a) Precipitation anomalies in February 2011 for (a) the SINTEX-F1 prediction for the June 1st, 2010 initialization and (b) observational data of GPCP (mm day^−1^). The GrADS software was used for this figure.

In the following December 2010, the development phase of the 2011 warm event, the La Niña reached the maximum in the tropical Pacific. As the La Niña matured, the induced easterly wind anomalies over the equatorial western Pacific intensified the Indonesian Throughflow and the Leeuwin Current in austral summer^1^ through *the Clarke-Meyers effect*^14^,^15^. Those atmospheric and oceanic conditions were predicted very well 7 months in advance (Figs. 4c, d). In February 2011, northerly wind anomalies off the west coast of Australia associated with anomalous low sea level pressure above the southeastern Indian Ocean further accelerated the Leeuwin Current and the coastal downwelling, suggesting the existence of a coastal ocean-atmosphere positive feedback. The SINTEX-F1 successfully predicted northerly wind anomalies and SST anomalies off the west coast of Australia in February 2011 when initialized on June 1st, 2010 (Figs. 4e, f). In accord to the matured Ningaloo Niño, most part of Australia experienced wetter-than-normal condition. In particular, northwestern Australia received more precipitation above 5 mm day^−1^ in February 2011 (Fig. 5b). This was also reasonably predicted by the SINTEX-F1 (Fig. 5a). We need further research to explore possible links between the Ningaloo Niño and the Australian rainfall anomaly, which could also be influenced by La Niña^16^ and the Australian monsoon^17^.

**Figure 6:**
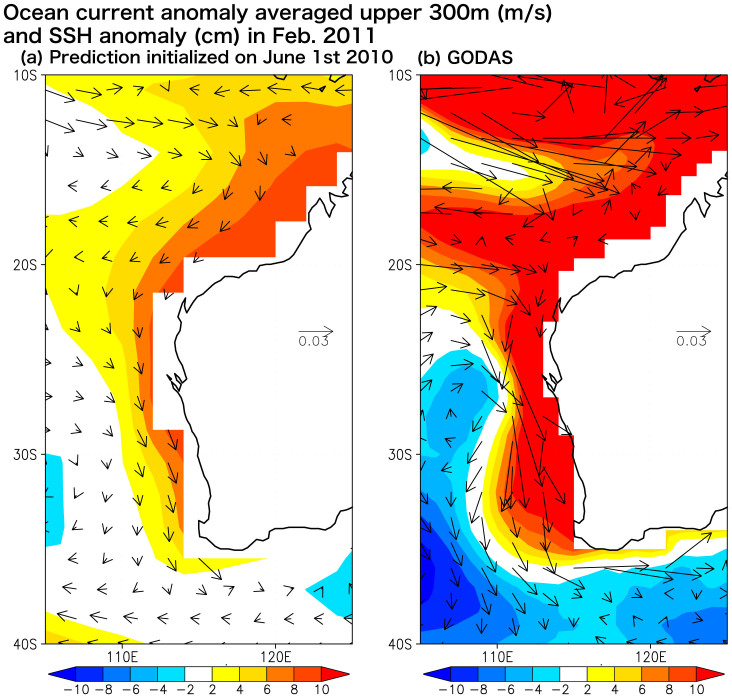
(a) Ocean current anomalies averaged above a depth of 300 m (vector; m s^−1^) and sea surface height anomalies (shaded; cm) in February 2011 for (a) the SINTEX-F1 prediction initialized on June 1st, 2010 and (b) the assimilation data of GODAS. The GrADS software was used for this figure.

Although the 2011 Ningaloo Niño prediction 2 seasons ahead was in good qualitative agreement with the observation, the predicted amplitude of the Ningaloo Niño was only 50% of that of the observed (Figs. 3 and 4). It may be due to the coarse horizontal resolution of the ocean component, which may have reduced the heat transport of the Leeuwin Current^1^,^10^ as well as the local ocean-atmosphere positive feedback. Fig. 6 shows the ocean current anomalies in February 2011. The SINTEX-F1 predicted the intensification of the Leeuwin Current qualitatively 2 seasons in advance, but its strength is only 30% of the assimilation data of GODAS. This underestimation may have affected the evolution of the Ningaloo Niño in its development phase (Fig. 3). Since the Leeuwin Current is an eastern boundary current strongly trapped off the west coast of the Australia within only a few degree, the zonal resolution of about 2° in the present ocean component may be too coarse to simulate its transport correctly^10^.

## Discussion

Using the SINTEX-F1 CGCM, we have examined its prediction skill of the newly discovered coastal climate mode off the west coast of the Australia, namely the Ningaloo Niño. It has turned out that the model is skillful in predicting the Ningaloo Niño (ACC > 0.6 and normalized RMSE < 1) up to 5-month lead when initialized on the first day of each month form May to November. In particular, the Ningaloo Niño is predicted very well when the model is initialized in austral winter-spring. Also, we have focused on the prediction of the unprecedented extreme warm event of the 2011 Ningaloo Niño. The SINTEX-F1 prediction initialized on June 1st, 2010 successfully predicted this extreme warm event in February 2011, i.e. 9 months in advance. The model reasonably predicted the rapid development of the La Niña condition, the easterly wind anomalies over the equatorial western Pacific in December 2010. It also predicated successfully warm SST anomalies off the west coast of Australia with northerly wind anomalies, the intensification of the Leeuwin Current, and the coastal downwelling in February 2011.

The Ningaloo Niño has serious impacts on marine ecosystems, but also it may influence on Australian summer precipitation through induced anomalous atmospheric circulations. As the 2011 Ningaloo Nino matured in February 2011, most part of Australia experienced wetter-than-normal condition, which was also reasonably predicted by the model initialized on June 1st, 2010. Further studies are necessary for understating the Ningaloo Niño's influences on Australian summer precipitation in more detail, because Australian climate is also influenced by the Australian Monsoon, El Niño/Southern Oscillation (ENSO), and Indian Ocean Dipole^16^,^17^,^18^,^19^,^20^,^21^,^22^. Possible roles of the Madden-Julian Oscillation may be also important^23^,^24^.

The potential source of the predictability of the 2011 Ningaloo Nino lies in the fact that the SINTEX-F1 reasonably predicts the quick transition from El Niño to La Niña in the tropical Pacific in late 2010 (Supplementary-Fig. 1). Although several other models predicted a neutral-state or a return of El Niño in late 2010, the SINTEX-F1 has correctly predicted the La Niña evolution (Supplementary-Fig. 2). Its success was widely reported in various newspapers in Japan, Australia, India and several other Southeast Asian countries^25^. Some ensemble forecast members which underestimated the La Niña condition in December 2010 are apt to fail to predict the Ningaloo Niño in February 2011 (Supplementary-Fig. 3). Hence, the successful prediction of the Ningaloo Niño may be due to the good prediction skill of ENSO by the SINTEX-F1 CGCM^26^,^27^,^28^. We also investigated the seasonal predictability of other extreme warm events of 1996/97 and 1999/2000, when the NNI was above two standard deviation in austral summer (Supplementary-Fig. 4). In Jan. 2000, the NNI was above two standard deviation, while the prediction from June 1st, 1999 shows a 56% (33%) chance of the warm event above one (two) standard deviation. Although the prediction underestimated the amplitude, similarly to the 2011 warm event, we may conclude that the 1999/2000 extreme event is also predictable qualitatively. Predictability of the 1999/2000 extreme event is also due to the occurrence of La Niña in the tropical Pacific and its high prediction skill. We note, however, that the 1996/97 warm event which occurred without La Niña event is not predictable by the SINTEX-F1 CGCM.

Although the SINTEX-F1 prediction of the 2011 Ningaloo Niño 2 seasons ahead is in good qualitative agreement with the observation, the predicted amplitude of the Ningaloo Niño is only 50% of the observed. The coarse horizontal resolution of the ocean component of the SINTEX-F1 may be not enough to simulate the Leeuwin Current and coastal downwelling off the west coast of Australia qualitatively. To minimize such a model bias, Hendon and Wang^10^ developed an empirical downscaling model for the Leeuwin Current, using the seasonal forecast outputs from a CGCM that captures the transmission of the large-scale sea level anomalies from the Pacific: *the Clarke-Meyers effect*. The model is skillful in predicting sea level anomalies off the west coast of Australia at 4–9 months lead due to the high prediction skill of ENSO. This is consistent with our results. Also, the coarse-resolution may fail to capture the local ocean-atmosphere feedback. Therefore, it is important to improve climate prediction models to capture such regional variations and thus to make seasonal climate information more beneficial to the regional societies. The current initialization method adopting a simple coupled SST-nudging initialization scheme may have limited the predictability, too. We expect that adoption of a suitable three-dimensional ocean data assimilation method will improve the preconditioning phase and the seasonal predictability of the Indian Ocean variations. We are developing the new high-resolution version of SINTEX-F2 GCM with new initialization scheme to challenge the improvement of the prediction skill for regional climate modes as typified by the Ningaloo Niño.

## Methods

### Ensemble seasonal prediction by SINTEX-F1 coupled GCM

The JAMSTEC seasonal prediction system was built on the basis of the Scale Interaction Experiment-Frontier (SINTEX-F1) fully coupled global ocean–atmosphere GCM^11^,^12^. The atmospheric component (ECHAM4.6) has a resolution of 1.18° (T106) with 19 vertical levels. The oceanic component (OPA8.2) has a relatively coarse resolution of a 28 Mercator horizontal mesh (about 2° × 2°) but with an equatorial intensification up to 0.58° in the meridional direction. It has 31 levels in the vertical from the surface to the bottom with a relatively fine resolution of 10 m from the sea surface to 110-m depth. The air–sea fluxes are exchanged every two hours without any corrections. Initial conditions for prediction are generated using a simple coupled SST-nudging initialization scheme. The prediction system has 27 ensemble members with uncertainties of both initial conditions and model coupling physics. When calculating model predicted anomalies, we have removed model climate drifts at each lead time in a posteriori manner using the hindcast outputs. We have calculated the ensemble mean by averaging 27 members simply. The real-time forecast results are available at http://www.jamstec.go.jp/frcgc/research/d1/iod/index.html. The SINTEX-F1 prediction system has demonstrated high performance in predicting ENSO and Indian Ocean Dipole^26^,^27^,^28^,^29^,^30^,^31^.

### Observational datasets

To evaluate the SINTEX-F1 prediction results, we use the NOAA OISSTv2^32^ for SST, the NCEP/NCAR reanalysis data^33^ for surface wind, the Global Precipitation Climatology Project dataset (GPCP^34^) for precipitation, and the Global Ocean Data Assimilation System (GODAS^35^) for ocean current upper 300 m. Monthly climatologies are calculated by averaging monthly data over 1983–2006, and then anomalies are defined as deviations from the monthly mean climatologies. For ocean heat content anomalies, we adopt the JAMSTEC-ARGO gridded dataset, which uses the monthly ocean subsurface temperature using data from Argo floats, the Triangle Trans-Ocean Buoy Network (TRITON), and available conductivity-temperature-depth (CTD) casts^36^.

## Author Contributions

T.D., S.B. and T.Y. contributed to designing the research, interpreting results, and writing the paper. T.D. performed the seasonal prediction system on a basis of the ocean-atmosphere coupled model and analyzed the observation data and model prediction outputs.

## Supplementary Material

Supplementary Information Supplementary Figures

## Figures and Tables

**Table 1:** The SINTEX-F1 probability prediction of the Ningaloo Niño Index in February 2011 for the June 1st, 2010 initialization

	Above 3σ	3 ~ 2σ	2 ~ 1σ	1 ~ −1σ	−1 ~ −2σ	−2 ~ −3σ	Below −3σ
(a) The number of ensemble member	3	9	11	4	0	0	0
(b) Probability prediction (%)	11	33	41	15	0	0	0

(a) The number of ensemble member categorized into multiple standard deviation of the observed NNI in 1983–2006 (σ), namely 0.72°C. Total is 27 members. The observed NNI in February 2011 is 2.4°C, then is categorized to “Above 3σ” (gray shaded).

(b) Probability prediction (%): values in (a) divided by 27.
