# Supplementary material for: Predictability of the Ningaloo Niño/Niña
Source: Sci Rep. 2013 Oct 8;3:2892. doi: 10.1038/srep02892 (PMC3792415; doi:10.1038/srep02892)
Supplement: Supplementary Information — Supplementary Figures [file srep02892-s1.doc]

**Supplementary Information for**

***“Predictability of the Ningaloo Niño/Niña”***

Takeshi Doi^1,2^, Swadhin K. Behera^1,2^, and Toshio Yamagata^2^

*^1^RIGC/JAMSTEC, Yokohama, Japan*

*^2^ Application Laboratory/JAMSTEC, Yokohama, Japan*

*Submitted to Scientific Reports*

*(2nd-round revised version)*

Sep 13, 2013

_____________________________________________________________________

*Corresponding author address: Takeshi Doi, Research Institute for Global Change (RIGC), Japan Agency for Marine-Earth Science and Technology (JAMSTEC), 3173-25 Showa-machi, Kanazawa-ku, Yokohama, 236-0001, Japan.*

*E-mail: takeshi.doi@jamstec.go.jp*

Supplementary-Fig.1


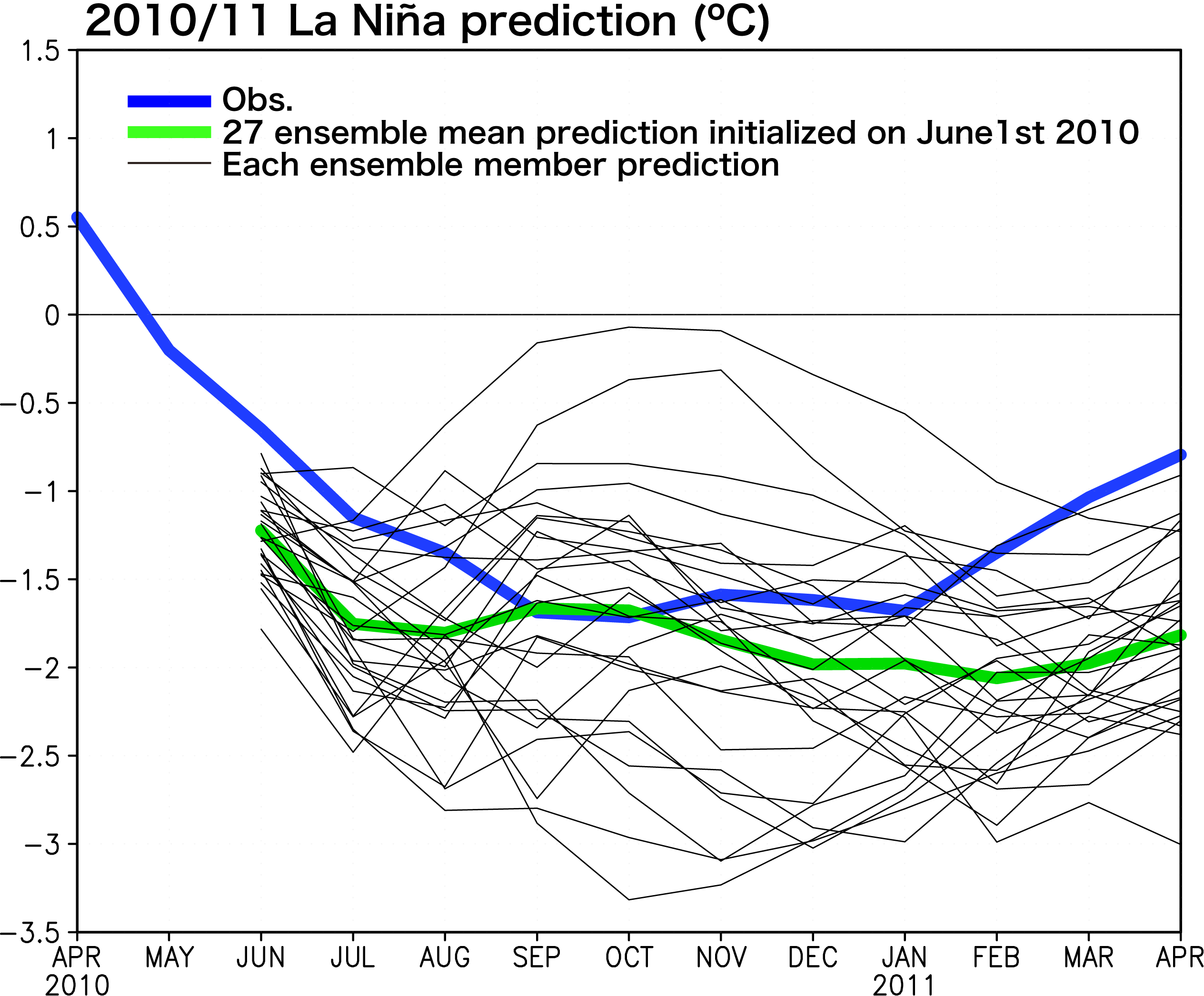


Supplementary-Fig. 1: Same as Fig.3, but for the Niño3.4 index (190º-240ºE, 5ºS-5ºN).

Supplementary-Fig.2


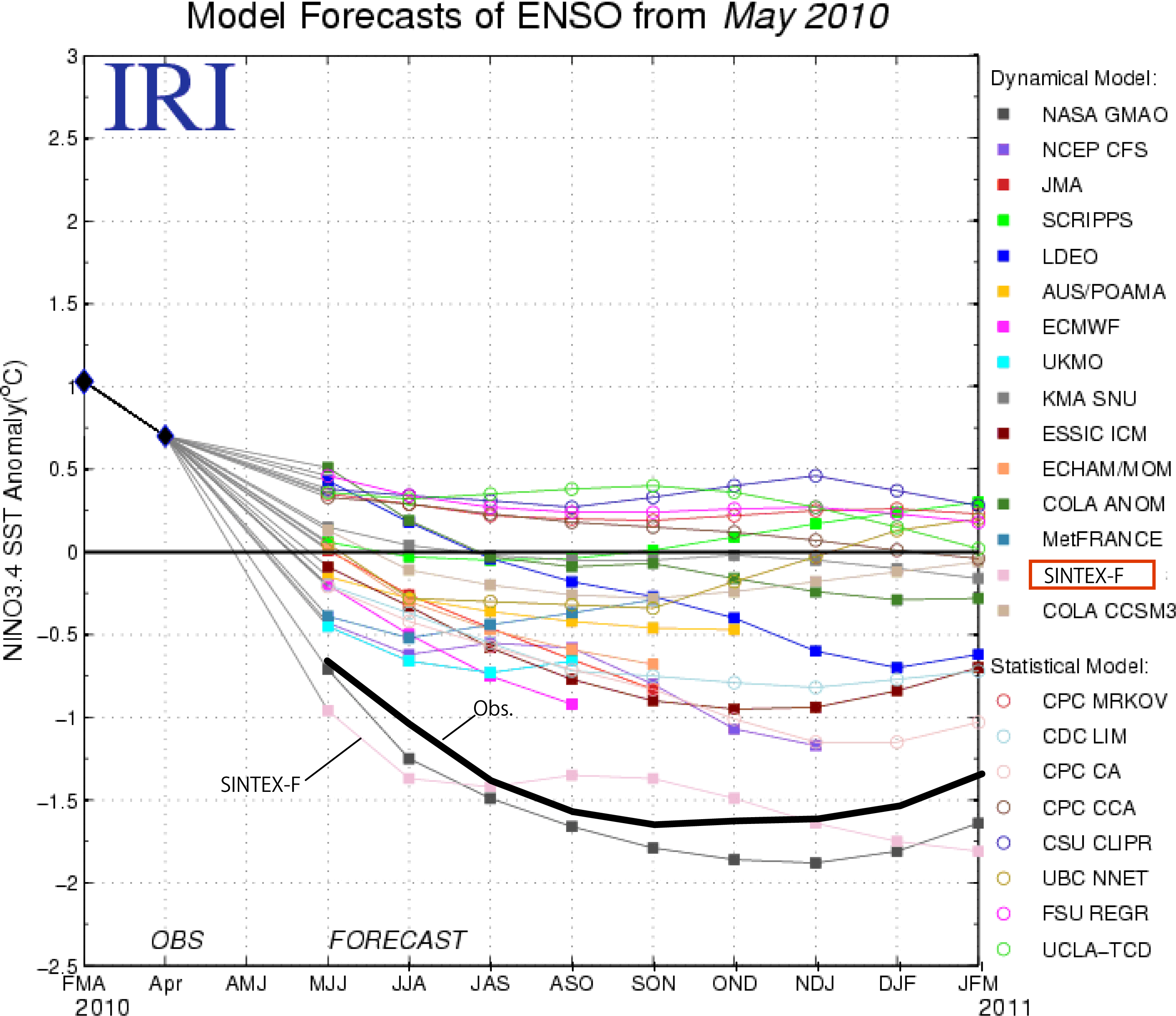


Supplementary-Fig.2: Model predictions of ENSO for the May 2010 initialization on an International Research Institute for Climate and Society (IRI) web page (available at http://portal.iri.columbia.edu/portal/server.pt?space=CommunityPage&control=SetCommunity&CommunityID=945&PageID=0). The SINTEX-F1 correctly predicted the strong La Niña condition in late 2010, although almost all of other models failed.

Supplementary-Fig.3


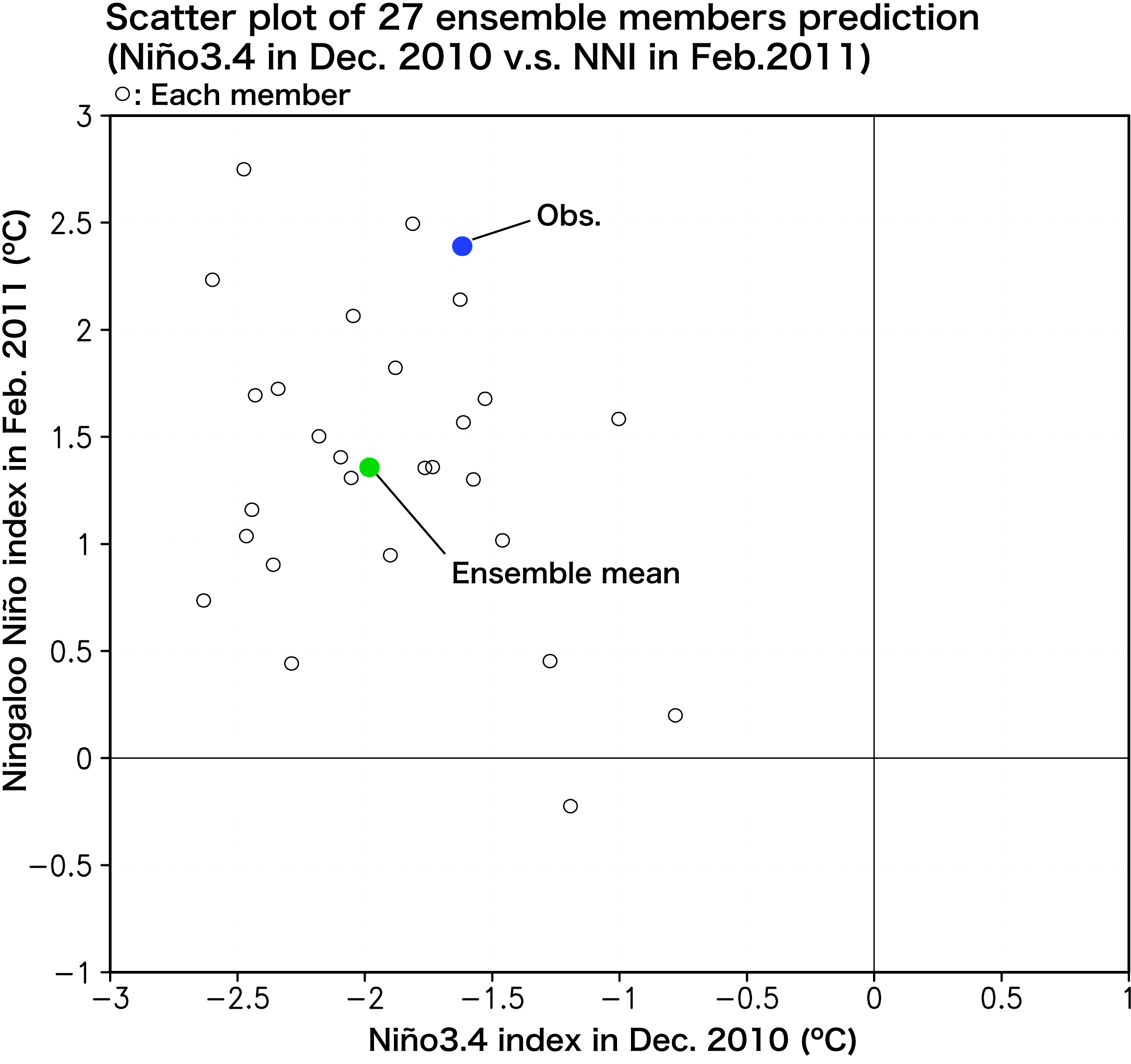


Supplementary-Fig.3: Scatter plot of 27-ensemble members prediction when initialized on June 1st, 2010 for the Niño3.4 index in December 2010 and the Ningaloo Niño index in February 2011 (ºC). Each member is shown by open circle. Ensemble mean is shown by green circle. Observational data of NOAA OISSTv2 is shown by blue.

Supplementary-Fig.4


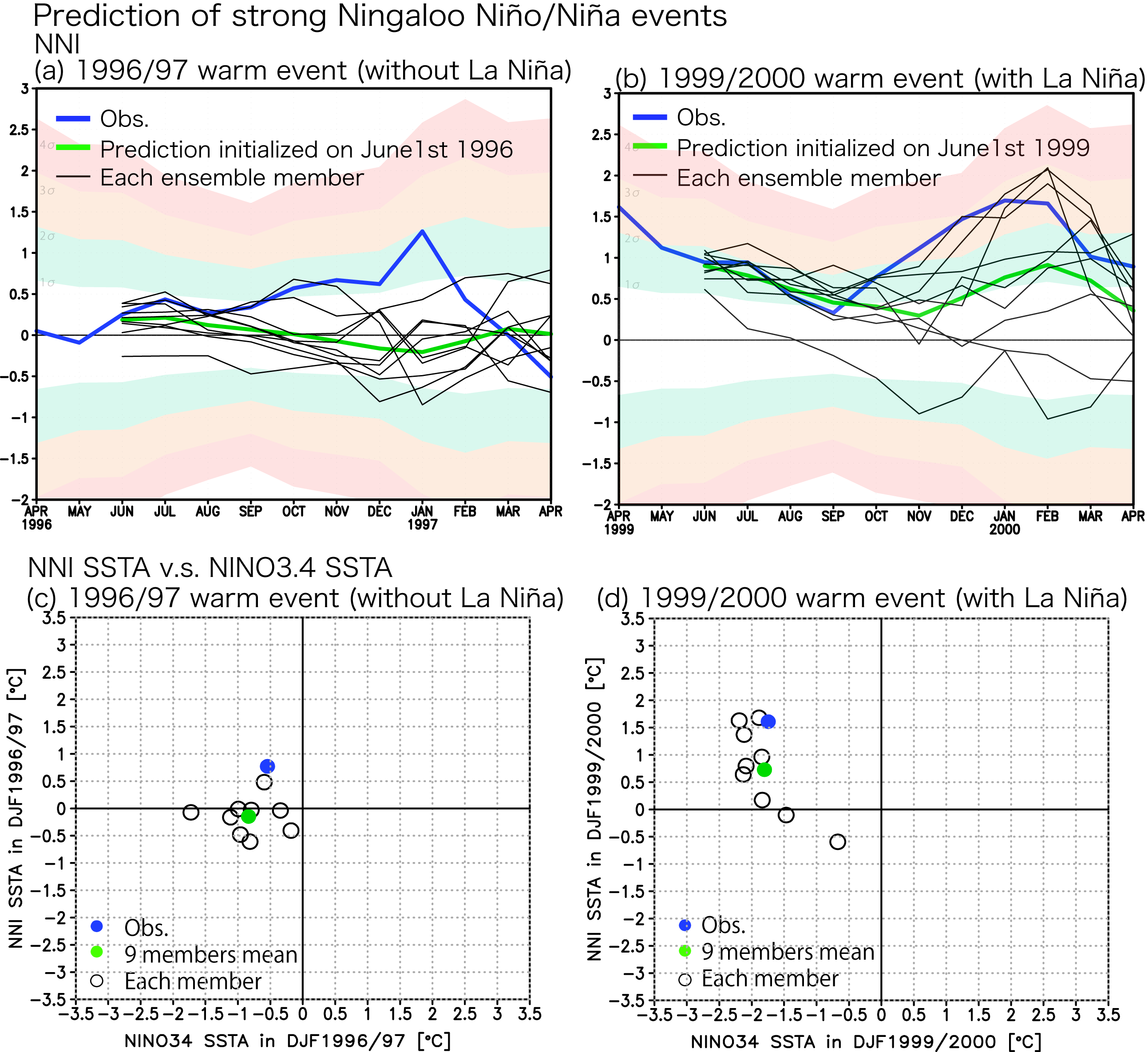


Supplementary-Fig.4: 9-ensemble member prediction of strong Ningaloo Niño events. (a) Same as Fig. 3, but for 1996/97 warm event. (b) Same as (a), but for 1999/2000 warm event. (c) Same as Supplementary-Fig.3, but for DJF1996/97. (d) Same as (c), but for DJF1999/2000.
